# ASK1‐Induced FGF21 Synthesis in the Liver Prevents Obesity in Mice

**DOI:** 10.1002/oby.70261

**Published:** 2026-07-16

**Authors:** Anne Goergen, Tenagne D. Challa, Carlos Villaroel‐Vicente, Marcela Borsigova, Pim P. van Krieken, Christian Wolfrum, Matthias Blüher, Stephan Wueest, Daniel Konrad

**Affiliations:** ^1^ Division of Pediatric Endocrinology and Diabetology University of Zurich Zurich Switzerland; ^2^ Children's Research Center, University Children's Hospital University of Zurich Zurich Switzerland; ^3^ Rosen Lab, Division of Endocrinology, Diabetes, and Metabolism Beth Israel Deaconess Medical Center Boston Massachusetts USA; ^4^ Institute of Food, Nutrition and Health ETH Zurich Schwerzenbach Switzerland; ^5^ Medical Department III ‐ Endocrinology, Nephrology, Rheumatology University of Leipzig Medical Center Leipzig Germany; ^6^ Helmholtz Institute for Metabolic Obesity and Vascular Research (HI‐MAG) of the Helmholtz Zentrum München at the University of Leipzig and University Hospital Leipzig Leipzig Germany; ^7^ Zurich Center for Integrative Human Physiology University of Zurich Zurich Switzerland

**Keywords:** adipose tissue, body weight, high fat diet, insulin resistance, liver

## Abstract

**Objective:**

Fibroblast growth factor 21 (FGF21) is a stress‐induced hormone secreted by the liver. It has a beneficial role in the regulation of glucose and energy metabolism. Herein, we identify apoptosis signal regulating kinase 1 (ASK1) as an inducer of hepatic FGF21 synthesis.

**Methods:**

Glucose and energy metabolism were assessed in liver‐specific ASK1 overexpressing (ASK1^+hep^) and control littermate (ASK1^f/f^) mice fed a regular chow or high fat diet (HFD) for 20 weeks. Liver and fat depots were analyzed using RNA sequencing. Hepatic *Fgf21* expression was downregulated using adeno‐associated virus (AAV) expressing short‐hairpin RNA sequences. *ASK1* and *FGF21* expression was determined in human liver samples.

**Results:**

HFD‐fed ASK1^+hep^ mice exhibited improved glucose metabolism, elevated energy expenditure, and reduced body weight. FGF21 plasma levels were increased in HFD‐fed ASK1^+hep^ mice, and its reduction in vivo blunted beneficial effects on obesity. Mechanistically, ASK1 promoted hepatic *Fgf21* gene expression via the transcription factor ATF4. In humans, hepatic *ASK1* expression correlated positively with *FGF21* and negatively with BMI and visceral fat area.

**Conclusions:**

We describe a novel role of hepatic ASK1 in the synthesis of FGF21. Hence, increasing hepatic *ASK1* expression might be a novel strategy to combat obesity‐associated metabolic complications.

## Study Importance

**What is already known?:**

- FGF21 is a stress‐induced hormone that is mainly secreted by the liver.
- FGF21 induces thermogenesis‐driven energy expenditure in adipose tissue and, thus, improves glucose metabolism.

**What does this study add?:**

- HFD‐fed liver‐specific ASK1 overexpressing mice exhibited improved glucose metabolism, elevated energy expenditure, and reduced adiposity.
- FGF21 plasma levels were highly increased in these mice and its reduction blunted beneficial effects on body weight gain and adiposity.
- In humans, hepatic *ASK1* expression correlated positively with *FGF21* and negatively with BMI and visceral fat area.

**How might these results change the direction of research or the focus of clinical practice?:**

- Increasing circulating FGF21 via induction of hepatic *ASK1* expression might be a novel strategy to combat obesity‐associated metabolic complications.

## 1 Introduction

The rising prevalence of overweight and obesity poses a heavy burden for societies and healthcare systems all over the world [1]. Obesity is often associated with metabolic comorbidities such as cardiovascular incidents, type 2 diabetes, and metabolic dysfunction‐associated steatotic liver disease (MASLD) [2, 3]. Importantly, cardiovascular and metabolic diseases are the primary causes of disability and death globally, impacting the quality of life for millions of people [4].

Fibroblast growth factor 21 (FGF21) is a stress‐induced hormone that is mainly secreted by the liver and has an important role in the regulation of glucose and energy metabolism [5]. FGF21 is also produced by brown adipose tissue (BAT), where it is induced by β‐adrenergic stimulation, cold exposure, and dietary interventions [6, 7, 8]. FGF21 protected mice from diet‐induced obesity and lowered blood glucose concentrations in diabetic mice [9, 10, 11], presumably via increased thermogenesis‐driven energy expenditure [12]. Indeed, it has been shown that FGF21 leads to enhanced energy expenditure via activation of BAT and induction of white adipose tissue (WAT) browning [6, 13]. Thermogenesis is mainly, but not exclusively, driven by uncoupling protein 1 (UCP1), a protein that uncouples the proton gradient over the inner mitochondrial membrane from ATP synthesis, thus creating a futile cycle that produces heat [14]. Endocrine FGF21 analogues have thus gained attention for their potential to directly target adipose tissue and the liver to treat obesity [9, 11, 15]. However, pharmacological studies have also revealed adverse effects of administering supraphysiological doses of exogenous FGF21 [16, 17], underlining the need to find alternative methods to elevate circulating FGF21 levels.

MASLD and its progression to metabolic dysfunction‐associated hepatic steatohepatitis (MASH) as well as liver fibrosis are important comorbidities of the current obesity pandemic and constitute the most frequent liver diseases worldwide [18, 19]. We previously reported that apoptosis signal‐regulating kinase 1 (ASK1) blunts the development of MASLD and liver fibrosis [20]. In particular, we demonstrated that hepatic ASK1 knockout impaired glucose metabolism and accelerated the development of fatty liver disease, hepatic inflammation, and liver fibrosis in high fat diet (HFD)‐fed mice. Conversely, liver‐specific ASK1 overexpressing mice (ASK1^+hep^) were protected from HFD‐induced hepatic lipid deposition, suggesting a protective role of liver‐expressed ASK1 in MASLD [20]. Herein, we unravel that ASK1^+hep^ mice are resistant to HFD‐induced obesity and associated metabolic derailments such as impaired glucose tolerance. In addition, we show that ASK1 reduces body weight gain in a FGF21‐dependent manner in mice and provide evidence for a protective role of liver‐expressed ASK1 in the development of obesity in humans.

## 2 Methods

### 2.1 Human Studies

The expression of *ASK1* and *FGF21* mRNA was measured in liver biopsies from lean study participants and patients who underwent open abdominal surgery for Roux‐en‐Y bypass, sleeve gastrectomy, explorative laparotomy, or elective cholecystectomy. Liver biopsies were taken during surgery, immediately snap‐frozen in liquid nitrogen, and stored at −80°C until further preparation. Measurement of body fat, tissue sample handling, and analysis of samples have been performed as described previously [21, 22]. All investigations have been approved by the ethics committee of the University of Leipzig (363–10‐13,122,010 and 017–12‐230,112) and were carried out in accordance with the Declaration of Helsinki. All study participants provided witnessed written informed consent before entering the study. Human *ASK1* and *FGF21* mRNA expression was measured by qRT‐PCR using an Assay‐on‐Demand gene expression kit (HsXY_m1; Applied Biosystems, Darmstadt, Germany), and fluorescence was detected on an ABI PRISM 7000 Sequence Detector (Applied Biosystems). *ASK1* and *FGF21* mRNA expression was calculated relative to the expression of 18S rRNA (Hs99999901_s1; Applied Biosystems).

### 2.2 Animal Husbandry and Experiments

Liver‐specific ASK1‐overexpressing (ASK1^+hep^) mice on a C57BL/6J background were generated in collaboration with the company PolyGene (Rümlang, Switzerland) [20]. In brief, murine ASK1 cDNA was cloned into a ROSA26‐based targeting vector including a lox‐stop‐lox cassette for conditional expression. After electroporation of the vector into ES cells, cells were injected into blastocysts and transferred into foster mice. ASK1 overexpression was induced by crossing mice homozygous for the target construct (ASK1^f/f^) to Alb‐Cre mice (B6.Cg‐Speer6‐ps1Tg(Alb‐cre)21Mgn/J) leading to Cre‐lox‐mediated excision of the stop cassette. All animal experiments were conformed to the Swiss animal protection laws and were approved by the Cantonal Veterinary Office in Zurich, Switzerland (Licenses ZH193/2017 and ZH103/2021). There was no protocol registered for this study. Male and female mice, aged 6 to 26 weeks, were used for studies. After transfer from the breeding facility, mice were allowed to acclimatize for 1 week. All experiments were performed with mice kept in a 12 h:12 h light:dark cycle (light phase starting at 7 a.m.) in a pathogen‐free animal facility. Cages were enriched with crinklets and gentle tunnel handling was used. Animals were monitored weekly (habitus, fur, activity, locomotion, eye symptoms, and body weight). Human endpoints were defined as signs of pain (humpback or closed eyes) or body weight loss > 10%. Group allocation regarding diet (chow vs. HFD) was based on the initial body weight (similar average body weight in both groups). Group size was determined based on previous experiments performed in our laboratory. Experimenters were not blinded to group allocations. Treatment order of mice was based on the random sequence of ear markings. Mice were housed two to five mice per cage, in individually ventilated cages. Each single animal was considered a biological unit. The ambient temperature in the animal facility was kept constant at 22°C and the animals were fed standard rodent (chow) diet or 59% HFD (E15772‐347, ssniff‐Spezialdiäten GmbH, Soest, Germany), with ad libitum access to food and water. HFD experiments started when mice were 6 weeks old and maintained for either 7 or 20 weeks. Tissue was collected in animals fasted for 5 h (from 8 a.m. to 1 p.m.). ARRIVE reporting guidelines were used [23].

### 2.3 Adeno‐Associated Virus‐Mediated Reduction of Circulating FGF21

Adeno‐associated virus (AAV) expressing four non‐silencing (AAV shCo; ssAAV‐8/2‐p3‐chI[4x(shm/rNS)]‐EGFP‐WPRE‐bGHp(A)) or *Fgf21*‐silencing (AAV shFgf21; ssAAV‐8/2‐p3‐chI[4x:sh(mFgf21)]‐EGFP‐WPRE‐bGHp(A)) short‐hairpin RNA sequences was intraperitoneally injected (10^11^ gc dissolved in 100 μL 0.9% NaCl) into 6‐week‐old ASK1^f/f^ and ASK1^+hep^ mice at the beginning of the HFD feeding period. AAVs under the transcriptional control of the synthetic liver‐specific promoter p3 [24] were produced by the Viral Vector Facility (VVF) of the Neuroscience Center Zurich (ZNZ).

### 2.4 Isolation of Primary Hepatocytes

Mice were euthanized by CO_2_ asphyxiation and the abdominal cavity opened to expose the vena cava inferior. The latter was cannulated with a catheter and the liver perfused with 1× HBSS supplemented with 0.05mM EDTA for 4 min, followed by digestion using DMEM (glucose 1 g/L), containing 1% P/S, 15mM HEPES, and 32 μg/L Liberase for 4 min. The liver was removed from the mouse and cells released from the liver into ice‐cold DMEM (glucose 1 g/L) supplemented with 10% FBS and 1% P/S (full medium) until only connective tissue was left. The cells were filtered through a 100 μm filter and washed three times in full medium with intermittent centrifugations at 50*g*, 4°C and 2 min. Primary hepatocytes were isolated from the remaining cells by centrifugation with 90% Percoll and viable cells plated in plating medium at a density of 2*10^5^ cells/mL as described [25].

### 2.5 Inhibition of Stress Kinases and siRNA‐Mediated Knockdown of ATF4

After plating, primary hepatocytes were allowed to attach and recover for 3 h. Before adding inhibitors/transfection reagents, plating medium was replaced by maintenance medium (Williams E medium, prepared as described in [25]). Thereafter, JNK inhibitor (2 μM in DMSO; SP600125, Cat #10.1033, Focus Biomolecules, Plymouth Meeting, PA, USA), p38 inhibitor (2 μM in DMSO; SB203580, BML‐EI286 ENO Life Sciences, Farmingdale, NY, USA), or vehicle control (DMSO, 0.1%) was added. For RNA interference, Opti‐Mem and 200nM small interfering RNA (siRNA) targeting ATF4 (siATF4; ON‐TARGETplus Mouse Atf4 siRNA, L‐042737‐01‐0005, Horizon Discovery Biosciences Limited, Cambridge, UK) or control siRNA (siCtrl; ON‐TARGETplus Non‐targeting Pool, D‐001810‐10‐05, Horizon Discovery Biosciences Limited) was mixed and primary hepatocytes were transfected using Lipofectamine 2000 with antibiotic‐free maintenance medium. After 24 h, medium was aspirated and cells were washed with ice‐cold PBS and frozen at −80°C until further processing.

### 2.6 RNA Isolation and Quantitative RT‐PCR

Total RNA was extracted with the NucleoSpin RNA Set for NucleoZOL (Macherey‐Nagel, Düren, Germany). RNA concentration was determined spectrophotometrically using NanoDrop (ThermoFisher Scientific, Waltham, MA, USA). RNA was reverse transcribed with the GoScriptTM Reverse Transcription System (Promega, Madison, WI, USA). The following probes/primers were used: Ask1, Mm00434883_m1; Atf4, Mm00515324_m1; Fgf21, Mm00840165_m1; Fgfr, Mm00438930_m1, Klb, Mm00473122_m1, Gapdh, Mm99999915_g1 (Applied Biosystems, Rotkreuz, Switzerland). Relative gene expression values were obtained after normalization to Gapdh using the 2^−ΔΔCt^ method [26].

### 2.7 RNA Sequencing and Transcription Factor Enrichment Analysis

RNA sequencing was conducted by Novogene (Cambridge, UK). mRNA was purified using poly‐T oligo‐attached magnetic beads. cDNA synthesis was performed using random hexamer primers, followed by library preparation with specific protocols for directional and non‐directional libraries, including end repair, A‐tailing, adapter ligation, size selection, and amplification. Libraries were quantified using Qubit, real‐time PCR, and bioanalyzer before pooling and sequencing on Illumina platforms. Raw fastq data were processed to obtain clean reads by removing adapters and low‐quality reads and calculating Q20, Q30, and GC content. Clean reads were mapped to the reference genome using Hisat2, with read counts quantified by FeatureCounts and gene expression estimated as FPKM. Differential expression analysis was conducted with DESeq2 for biological replicates and edgeR for non‐replicates, applying Benjamini‐Hochberg corrections. Genes with adjusted *p* ≤ 0.05 were deemed differentially expressed. Transcription factor analysis was performed using ChEA3 to identify enriched transcription factors [27].

### 2.8 Western Blot Analysis

Tissues were lysed in ice‐cold lysis buffer containing 150mM NaCl, 50mM Tris–HCl (pH 7.5), 1mM EGTA, 1% NP‐40, 0.25% sodium deoxycholate, 1mM sodium pyrophosphate, 1mM sodium vanadate, 1mM NaF, 10mM sodium β‐glycerolphosphate, 0.2mM PMSF, and a 1:1000 dilution of protease inhibitor cocktail (Sigma‐Aldrich, St. Louis, MO, USA). Protein concentration was determined using a BCA assay (Pierce, Rockford, IL, USA). Equal amounts of protein were resolved by sodium dodecyl sulfate polyacrylamide gel electrophoresis (SDS‐PAGE) and electrotransferred onto nitrocellulose membranes (0.2 μm, BioRad, Reinach, Switzerland). Equal protein loading on membranes was checked by Ponceau S staining. Blots were blocked in tris‐buffered saline (50mM Tris–HCl, 150mM NaCl) containing 0.1% Tween (TBS‐T) supplemented with 5% nonfat dry milk. Membranes were then placed in a 50 mL Falcon tube and incubated overnight at 4°C with gentle rotation with the respective primary antibody solutions. Antibody–antigen complexes were detected by using the ECL system and visualized with the ChemiDoc MP Imaging System (BioRad). The following primary antibodies were used: UCP1, PA1‐24894 (ThermoFisher Scientific; diluted 1:1000); HSP90, 4877 (Cell Signaling, Danvers, MA, USA; diluted 1:1000). Protein levels were quantified using the Image Lab software (BioRad, version 5.2.1) and normalized to HSP90.

### 2.9 Circulating FGF21 and Insulin Levels

Plasma FGF21 and insulin levels were measured using the kits Mouse and Rat FGF21 ELISA (BioVendor (Brno, Czech Republic); intra‐assay coefficients of variation [CV] 8.4%, inter‐assay CV 8.7%) and the Insulin Mouse Ultra Sensitive ELISA (Chrystal Chem (Itasca, IL, USA); intra and inter‐assay CV < 10%), respectively.

### 2.10 Indirect Calorimetry

Mice were placed individually in airtight cages designed for metabolic phenotyping in an open‐circuit indirect calorimetric system (PhenoMaster, TSE Systems, Bad Homburg, Germany) for 4 days. A total of 72 data points (3 per hour) for food intake, O_2_ consumption, and CO_2_ production were recorded over every 24‐h period. Energy expenditure was calculated using the manufacturer's software. The average of Days 2 to 4 was used for data calculation, as body weight remained ±stable during these days.

### 2.11 Insulin and Glucose Tolerance Test

For intraperitoneal glucose tolerance test, mice were fasted overnight (from 5 pm to 8 am) and for intraperitoneal insulin tolerance tests for 3 h (from 9 a.m. to 12 p.m.). Glucose (2 g/kg body weight) or human recombinant insulin (1.0 U/kg body weight) was injected intraperitoneally (0.30 mm [30G] × 8 mm; BD Micro‐Fine, Becton Dickinson, Franklin Lakes, NJ, France). Blood glucose concentration was measured with a glucometer (Accu‐Check Aviva, Roche Diagnostik, Rotkreuz, Switzerland) collected from tail vein incisions at 0, 15, 30, 45, 60, 90, and 120 min.

### 2.12 Data Analysis

All results are expressed as mean ± standard error of the mean (SEM). Statistical analysis was performed using two‐tailed, unpaired Student's *t*‐test, ANCOVA (energy expenditure with body weight as a covariate), or two‐way ANOVA with Bonferroni or Tukey's multiple comparisons test (GraphPad Prism Software, San Diego, CA, USA; version 8.0.0), assuming normal distribution. If the data were not normally distributed (as assessed by Shapiro–Wilk test), Mann–Whitney test was performed. Outliers defined by the ROUT test were excluded from statistical analysis, a criteria that was established a priori. In human studies, linear relationships were assessed by Spearman correlation. A *p* value < 0.05 was considered to be statistically significant. GraphPad Prism was used for statistical analysis and to produce graphs; BioRender (https://www.biorender.com/) was used to create figures.

## 3 Results

### 3.1 ASK1 ^+hep^ Mice Are Resistant to HFD‐Induced Obesity

**FIGURE 1:**
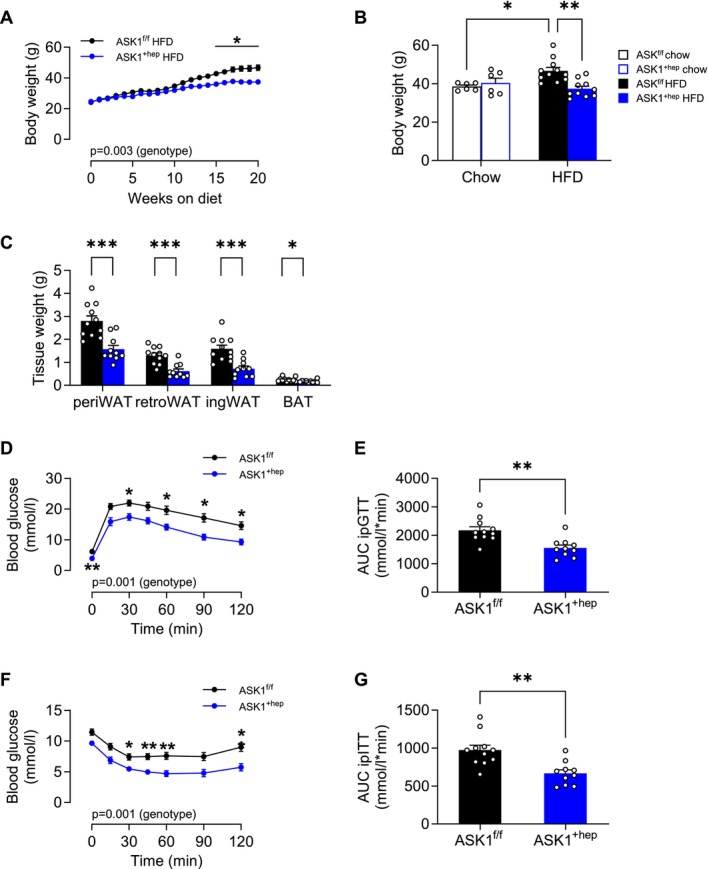
ASK1^+hep^ mice are resistant to HFD‐induced obesity. (A, B) Body weight development during (*p < 0.05) and at the end of 20 weeks of HFD in chow‐ and HFD‐fed (*p = 0.0168, **p = 0.0012) ASK1^f/f^ and ASK1^+hep^ mice (*n* = 10–11 mice for HFD and *n* = 6 for chow). (C) WAT depot weight (perigonadal [peri, ***p = 0.0004], retroperitoneal [retro, ***p = 0.0004], inguinal [ing, ***p = 0.0005]) and BAT (***p = 0.0472) of 20 weeks HFD‐fed ASK1^+hep^ (blue bar) and ASK1^f/f^ (black bar) (*n* = 10–11 mice). (D) Intraperitoneal glucose tolerance test (ipGTT) (*p < 0.05, **p < 0.01) including (E) area under curve (AUC) (**p = 0.0014) and (F) insulin tolerance test (ipITT) (*p < 0.05, **p < 0.01) including (G) AUC (**p = 0.0014) in ASK1^+hep^ and ASK1^f/f^ mice fed a HFD for 20 weeks (*n* = 10–11 mice). Data are shown as mean ± SEM. Statistical tests used: two‐way ANOVA for panels A, B, D, and F, Mann–Whitney test for panel C (retro), Student's *t* test for panels C (peri, ing, BAT), E, and G. [Color figure can be viewed at wileyonlinelibrary.com]

We have previously shown that liver‐specific ASK1 overexpressing (ASK1^+hep^) mice are partly protected from HFD‐induced hepatic steatosis [20]. As intended, ASK1^+hep^ mice revealed elevated ASK1 protein levels in the liver but not in other metabolic tissues when compared to control littermates (ASK1^f/f^). Moreover, hepatic phospho c‐Jun N‐terminal kinase (pJNK) protein concentrations were significantly increased in ASK1^+hep^ mice, indicating functional upregulation of the ASK1 pathway [20]. To elucidate the role of liver‐specific ASK1 overexpression beyond its protective role in the development of MASLD, we studied the effect of long‐term HFD feeding on glucose and energy metabolism in ASK1^+hep^ mice. To this end, male ASK1^+hep^ and ASK1^f/f^ mice were fed a regular chow or HFD for 20 weeks. While body weight gain in chow‐fed mice was similar between the genotypes (Figure S1A), HFD‐fed ASK1^+hep^ mice gained significantly less weight compared to control littermates, leading to ~10 g lower body weight after 20 weeks (Figure 1A). Importantly, HFD‐fed ASK1^+hep^ mice displayed similar body weight compared to chow‐fed animals after 20 weeks (Figure 1B), indicating that they were protected from HFD‐induced obesity. Moreover, weight of BAT and different WAT depots was significantly reduced (Figure 1C; see Figure S1B for data normalized to body weight), suggesting that ASK1^+hep^ mice are resistant to HFD‐induced adiposity. Similarly, liver weight was lower in HFD‐fed ASK1 overexpressing mice (2.0 ± 0.2 g in ASK1^f/f^ vs. 1.5 ± 0.1 g in ASK1^+hep^ mice, *p* = 0.002), which may be contributing to reduced body weight in these mice. In parallel, HFD‐fed ASK1^+hep^ mice revealed significantly improved glucose tolerance (Figure 1D,E) as well as ameliorated insulin sensitivity (Figure 1F,G) compared to ASK1^f/f^ mice. Consistently, HFD‐fed ASK1^+hep^ mice showed lower fasting blood insulin and glucose concentrations compared to littermate controls (Figure S1C,D). In contrast, glucose tolerance and insulin sensitivity at the age of 26 weeks were similar between chow‐fed ASK1^f/f^ and ASK1^+hep^ mice (Figure S1E,F). To summarize, liver‐specific ASK1 overexpression protects mice from HFD‐induced body weight gain and associated deterioration in glucose tolerance and insulin sensitivity.

### 3.2 Increased Energy Expenditure in HFD‐Fed ASK1 ^+hep^ Mice

**FIGURE 2:**
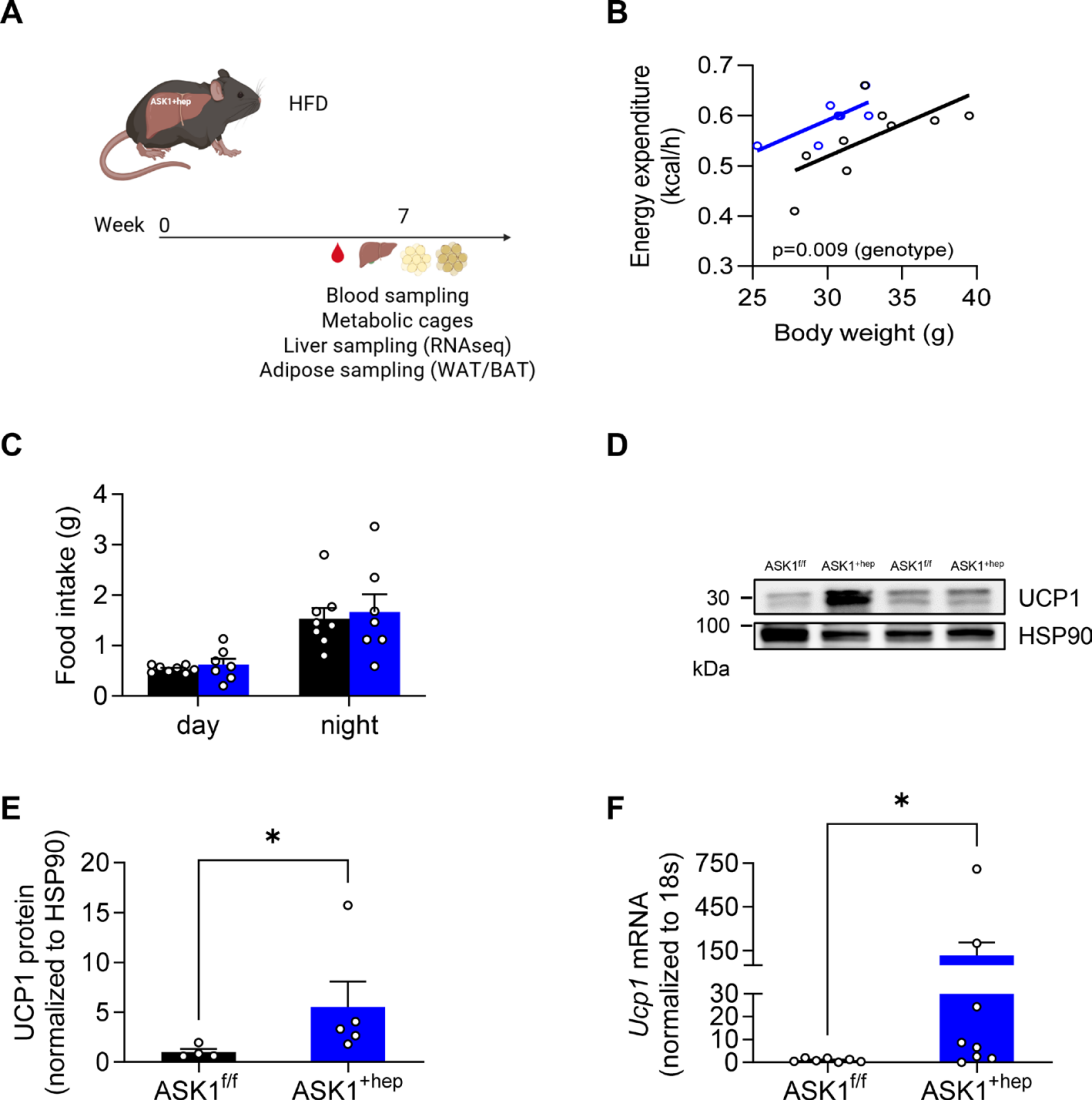
Increased energy expenditure in HFD‐fed ASK1^+hep^ mice. (A) Scheme of experimental procedures performed in mice fed a HFD for 7 weeks. (B) Linear regression analysis of energy expenditure in HFD‐fed ASK1^+hep^ and ASK1^f/f^ mice as a function of body mass (*n* = 8–9 mice). (C) Food intake in ASK1^+hep^ (blue bars) and ASK1^f/f^ (black bar) mice on HFD for 7 weeks during light phase and dark phase (*n* = 7–8 mice). (D) Representative Western blot of UCP1 and HSP90 in BAT of ASK1^f/f^ and ASK1^+hep^ mice. (E) Protein quantification of UCP1 (normalized to HSP90) in BAT of ASK1^+hep^ (blue bars) and ASK1^f/f^ (black bars) mice (*n* = 4–5 mice). *p = 0.0317. (F) mRNA expression of *Ucp1* in inguinal WAT of ASK1^+hep^ (blue bars) and ASK1^f/f^ (black bars) mice (*n* = 7–8). *p = 0.0289. Data are shown as mean ± SEM. Statistical tests used: ANCOVA for panel B, Mann–Whitney test for panel E and F. [Color figure can be viewed at wileyonlinelibrary.com]

Next, we sought to understand why ASK1^+hep^ mice are protected from HFD‐induced body weight gain. To this end, we analyzed whole body physiology and molecular composition of metabolic tissue in mice after 7 weeks of HFD (Figure 2A), a time point when body weight started to deviate between the two genotypes (Figure 1A). From a thermodynamic point of view, reduced body weight may originate from decreased food intake or increased energy expenditure. Thus, these parameters were assessed in metabolic cage units. Linear regression analysis (ANCOVA) revealed a significant body weight‐independent increase in energy expenditure in HFD‐fed ASK1^+hep^ mice (Figure 2B; see Figure S2A for bar and diurnal graphs), while food intake was not different between the genotypes (Figure 2C), indicating that reduced body weight gain in HFD‐fed ASK1^+hep^ mice originates from increased energy expenditure. Of note, locomotor activity was not increased in HFD‐fed ASK1^+hep^ compared to ASK1^f/f^ mice (Figure S2B), suggesting that increased thermogenesis rather than elevated locomotion increased energy expenditure in overexpressing mice. In support, levels of UCP1 were increased in BAT and WAT of ASK1^+hep^ mice after 7 weeks of HFD (Figure 2D–F), paralleled by decreased BAT whitening in ASK1^+hep^ mice (Figure S2C).

### 3.3 Elevated FGF21 Plasma Concentrations in HFD‐Fed ASK1 ^+hep^ Mice

**FIGURE 3:**
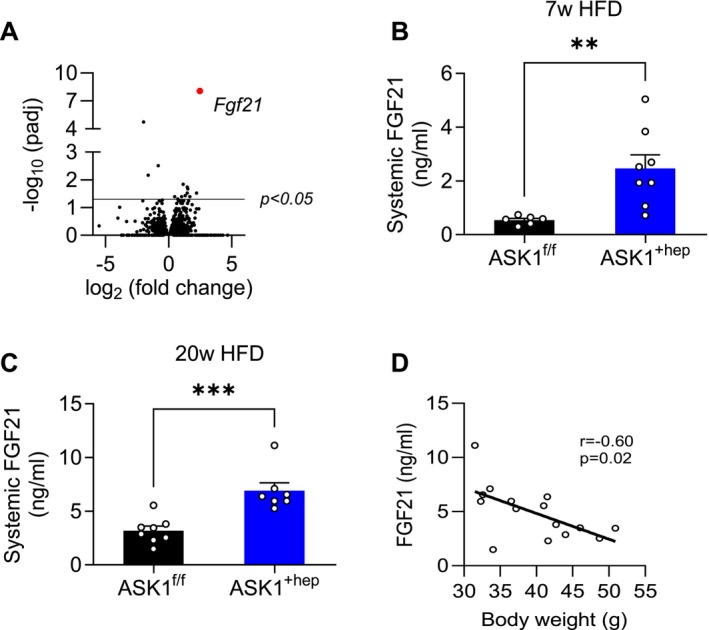
Elevated FGF21 plasma concentrations in HFD‐fed ASK1^+hep^ mice. (A) Volcano plot of hepatic RNA sequencing data of ASK1^+hep^ and ASK1^f/f^ mice after 7 weeks on HFD (*n* = 5 mice per group). (B) Circulating FGF21 levels in HFD‐fed ASK1^+hep^ (blue bars) and ASK1^f/f^ (black bars) mice after 7 weeks on HFD (*n* = 6–8 mice per group). **p = 0.0063. (C) Circulating FGF21 levels in HFD‐fed ASK1^+hep^ (blue bars) and ASK1^f/f^ (black bars) mice after 20 weeks on HFD (*n* = 7–8). ***p = 0.0006. (D) Correlation between body weight and circulating FGF21 plasma levels in 20 weeks HFD‐fed ASK1^+hep^ and ASK1^f/f^ mice (*n* = 15 mice). Data are shown as mean ± SEM. Statistical tests used: Student's *t* test for panel B, Mann–Whitney test for panel C, Pearson correlation for panel D. [Color figure can be viewed at wileyonlinelibrary.com]

Next, we performed bulk RNA sequencing of liver samples to identify potential hepatokines that mediate increased energy expenditure in HFD ASK1^+hep^ mice (Figure 2A). At necropsy after 7 weeks of HFD, WAT and body weight were not significantly different between the two genotypes (Figure S3A,B), confirming results from previous cohorts (Figure 1A). Importantly, differential gene expression analysis revealed *Fgf21* to be highly upregulated in livers of ASK1^+hep^ mice (Figure 3A) and was accompanied by a 4.6‐fold increase in circulating FGF21 levels in 7‐week HFD‐fed ASK1^+hep^ mice (Figure 3B). Conversely, expression of β‐klotho (*Klb*) and FGF receptor 1 (*Fgfr1*) was unaltered in BAT, WAT, and liver (Figure S3C–E). Similarly, expression of *Ask1* did not differ in WAT and BAT between ASK1^f/f^ and ASK1^+hep^ mice (Figure S3D,E). In line with data after 7 weeks of HFD, plasma FGF21 concentrations remained significantly increased after 20 weeks of HFD in ASK1^+hep^ mice (Figure 3C), and they correlated negatively with body weight (Figure 3D). While circulating FGF21 levels were ~30% higher in male chow‐fed ASK1^+hep^ mice compared to littermate controls, such differences were not significant (Figure S3F). Of note, circulating FGF21 concentrations were increased and body weight gain reduced in HFD‐fed female ASK1^+hep^ mice (Figure S3G,H), indicating a sex‐independent effect of ASK1. Taken together, increased energy expenditure in HFD‐fed ASK1^+hep^ mice is associated with elevated circulating FGF21 concentrations, suggesting that the latter may contribute to reduced body weight gain in these mice.

### 3.4 Increased *FGF21* Expression in HFD‐Fed ASK1 ^+hep^ Mice Is Mediated by ATF4

**FIGURE 4:**
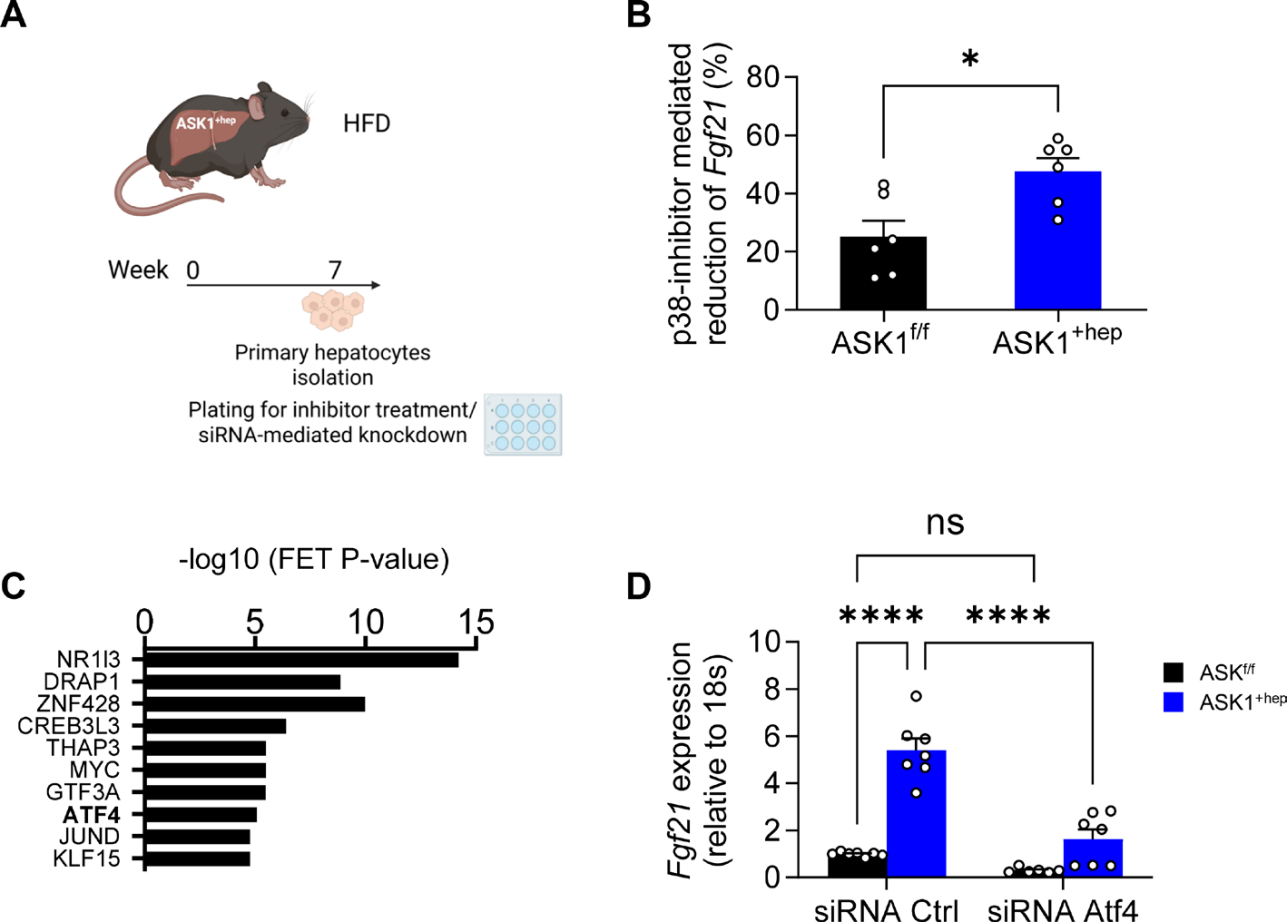
Increased *FGF21* expression in HFD‐fed ASK1^+hep^ mice is mediated by ATF4. (A) Scheme of experimental design after isolation of primary hepatocytes from HFD‐fed ASK1^fl/fl^ and ASK1^+hep^ mice. (B) Percent reduction of *Fgf21* gene expression after p38 inhibitor treatment of primary hepatocytes isolated from 7 weeks HFD‐fed ASK1^+hep^ (blue bar) and ASK1^f/f^ (black bar) mice (*n* = 6 cell culture wells from two independent experiments with one mouse per genotype and three technical replicates per condition). *p = 0.0109. (C) Top 10 enriched transcription factors in 7 weeks HFD‐fed ASK1^+hep^ vs. ASK1^f/f^ livers. Data are presented as −log10 of the Fisher's exact test (FET) *p* value. (D) *Fgf21* gene expression after siRNA‐mediated knockdown of ATF4 in primary hepatocytes isolated from 7 weeks HFD‐fed ASK1^+hep^ and ASK1^f/f^ mice (*n* = 6–7 cell culture wells from three independent experiments with one mouse per genotype and two to three technical replicates per condition). ****p < 0.0001. Data are shown as mean ± SEM. Statistical tests used: Student's *t* test for panel B, two‐way ANOVA for panel D. [Color figure can be viewed at wileyonlinelibrary.com]

ASK1 is a serine/threonine protein kinase and a member of the MAPK kinase kinase (MAP3Ks) family. Upon activation, ASK1 undergoes homodimerization and autophosphorylation and subsequently induces phosphorylation and activation of downstream kinases such as c‐Jun N‐terminal kinase (JNK) and p38 MAPK [28, 29, 30]. To investigate whether these kinases contribute to increased hepatic *Fgf21* expression in ASK1^+hep^ mice fed a HFD for 7 weeks, experiments in primary hepatocytes treated with JNK or p38 inhibitors were performed (Figure 4A). p38 inhibitor treatment reduced *Fgf21* transcript levels to a significantly higher extent in ASK1^+hep^ as compared to ASK1^f/f^ derived primary hepatocytes (Figure 4B and Figure S4A). In contrast, inhibiting JNK in ASK1^+hep^ hepatocytes did not reduce *Fgf21* expression levels (Figure S4B). Of note, JNK inhibition increased *Fgf21* expression in ASK1^f/f^ hepatocytes, indicating that JNK is a negative regulator of *Fgf21* transcription. In line, hepatic JNK signaling was found to suppress *Fgf21* expression [31]. This data indicate that p38 rather than JNK mediates the effect of ASK1 overexpression on *Fgf21* transcription in hepatocytes. To identify potential transcription factors that mediate the effect of the ASK1‐p38 axis on the expression of *Fg21*, we performed a transcription factor enrichment analysis using liver RNA sequencing data (Figure 3A), using ChIP‐X Enrichment Analysis 3 (ChEA3). ChEA3 is a computational tool that ranks transcription factors associated with user‐submitted gene sets based on evidence from multiple experimental and computational datasets [27]. As shown in Figure 4C, transcription factor 4 (ATF4) emerged as one of the top hits, indicating that its transcriptional activity may be elevated in livers of HFD‐fed ASK1^+hep^ mice. Importantly, ATF4 can be activated by p38 [32]. Moreover, recent evidence indicates that the stress‐induced increase in *Fgf21* expression is mediated by ATF4 [33, 34]. To test the hypothesis that ATF4 is mediating ASK1‐p38‐induced *Fgf21* expression, ATF4 was depleted in primary hepatocytes isolated from HFD‐fed ASK1^f/f^ and ASK1^+hep^ mice. To this end, hepatocytes were treated with scrambled siRNA or siRNA targeting ATF4. As expected, siRNA‐mediated knockdown of ATF4 significantly downregulated *Atf4* expression in both genotypes (Figure S4C). Moreover, increased *Ask1* expression in ASK1^+hep^ mice (Figure S4D) was paralleled by significantly increased *Fgf21* transcription in hepatocytes treated with scrambled siRNA (Figure 4D). Importantly, reduced *Atf4* expression significantly downregulated *Fgf21* in hepatocytes derived from ASK1^+hep^ but not from ASK1^f/f^ mice (Figure 4D), indicating that ATF4 plays a crucial role in the upregulation of *Fgf21* expression in ASK1 overexpressing hepatocytes.

### 3.5 Lowering Circulating FGF21 Abolishes Reduced Body Weight Gain in ASK1 ^+hep^ Mice

**FIGURE 5:**
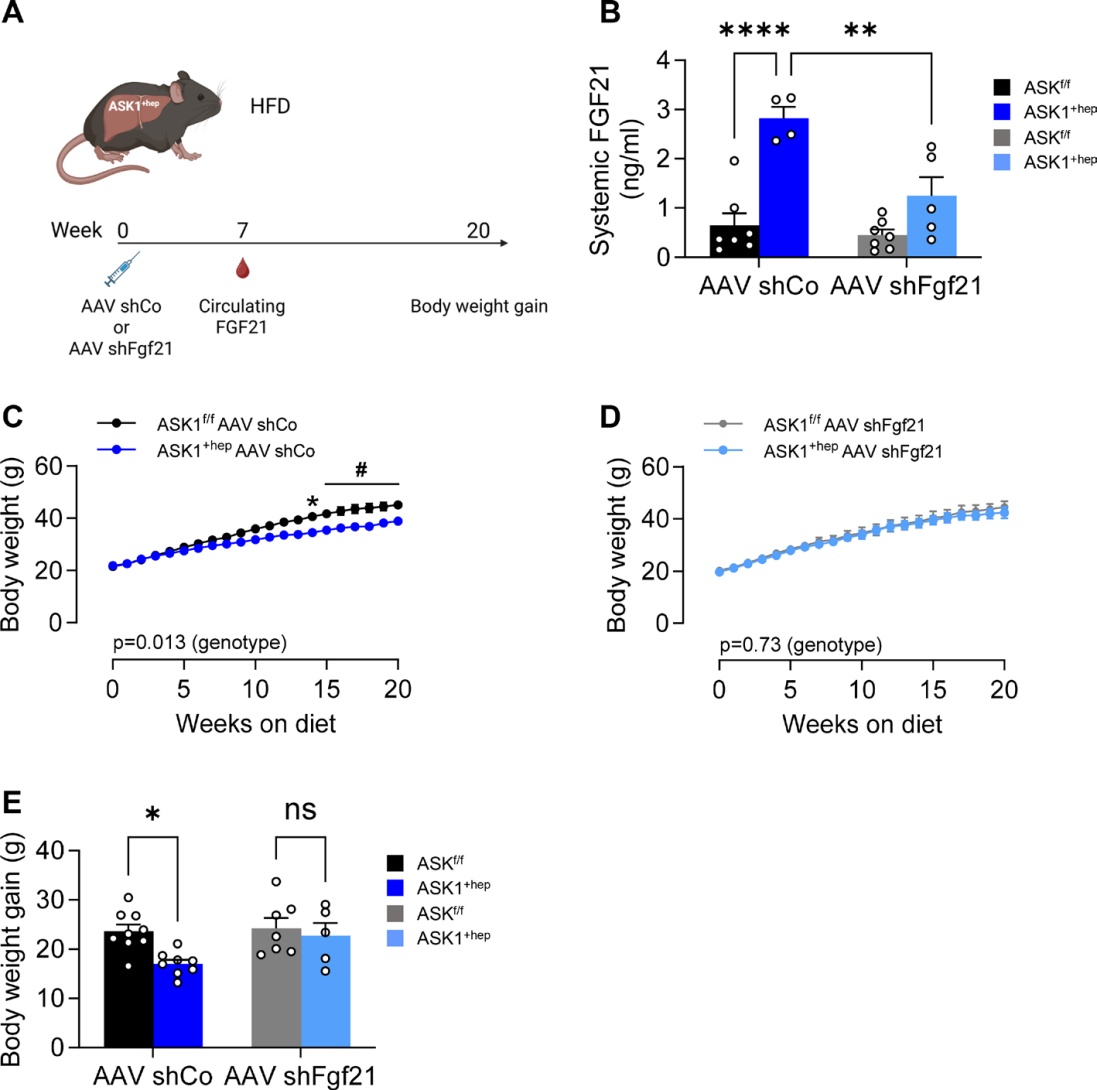
Lowering circulating FGF21 abolishes reduced body weight gain in ASK1^+hep^ mice. (A) Scheme of experimental procedures performed in mice injected with AAV to reduce FGF21 synthesis. (B) Circulating FGF21 levels in ASK1^+hep^ (blue bars, *n* = 4–5 mice) and ASK1^f/f^ (gray/black bars; *n* = 7 mice) injected with AAV shCo or AAV shFgf21 and fed a HFD for 7 weeks. **p = 0.0037, ****p < 0.0001. (C, D) Body weight of HFD‐fed ASK1^+hep^ (*n* = 5–8 mice) and ASK1^f/f^ (*n* = 7–9 mice) injected with AAV shCo or AAV shFgf21 during the 20‐week HFD period. ^#^ *p* = 0.0518–0.0875, *p = 0.0463. (E) Body weight gain in HFD‐fed ASK1^+hep^ (*n* = 5–8 mice) and ASK1^f/f^ (*n* = 7–9 mice) injected with AAV shCo or AAV shFgf21 after 20 weeks of HFD feeding. *p = 0.0234. Data are shown as mean ± SEM. Statistical tests used: Two‐way ANOVA. [Color figure can be viewed at wileyonlinelibrary.com]

To evaluate whether increased circulating FGF21 concentrations critically contribute to reduced body and WAT weight in HFD‐fed ASK1^+hep^ mice, we aimed to reduce hepatic FGF21 synthesis using an adeno‐associated virus (AAV) expressing *Fgf21*‐silencing short‐hairpin RNA sequences (AAV shFgf21). As a control, an AAV expressing non‐silencing short‐hairpin RNA sequences (AAV shCo) was used (Figure 5A). While a > 4‐fold increase in circulating FGF21 concentrations was confirmed in ASK1^+hep^ mice receiving AAV shCo after 7 and 20 weeks of HFD, AAV shFgf21 injection significantly reduced circulating FGF21 in ASK1^+hep^ mice (Figure 5B and Figure S5A). ASK1^+hep^ mice treated with AAV shCo gained significantly less weight compared to controls (Figure 5C) and revealed reduced WAT mass in different depots after 20 weeks of HFD (Figure S5B), confirming that liver‐specific ASK1 overexpression reduces HFD‐induced body weight and adiposity (Figure 1A,C). Importantly, ASK1^+hep^ mice treated with AAV shFgf21 were no longer protected from HFD‐induced weight gain (Figure 5D) and depicted similar WAT depot weight as ASK1^f/f^ mice after 20 weeks of HFD (Figure S5C). Analysis of body weight gain revealed that ASK1^+hep^ mice treated with AAV shCo gained less weight compared to ASK1^f/f^ mice, whereas AAV‐mediated blockade of FGF21 synthesis offset such differences (Figure 5E). These data suggest that elevated FGF21 levels in HFD‐fed ASK1^+hep^ mice are responsible for reduced body weight gain and adiposity.

### 3.6 Liver *ASK1* Expression Correlates Positively With *FGF21* but Negatively With Adiposity in Humans

**FIGURE 6:**
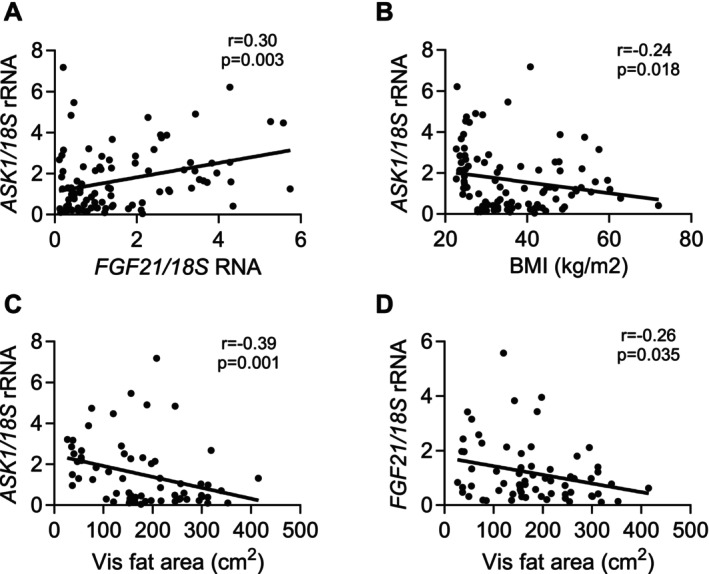
Liver *ASK1* expression correlates positively with *FGF21* but negatively with adiposity in humans. (A, B) Scatterplots of liver *ASK1* mRNA expression and liver *FGF21* mRNA expression (*n* = 96) or BMI (*n* = 97) in lean participants (*n* = 23) and people living with overweight/obesity (*n* = 74) with or without type 2 diabetes. (C, D) Scatterplot of liver *ASK1* (*n* = 66) or liver *FGF21* (*n* = 65) mRNA expression and visceral fat area (cm^2^) in lean participant (*n* = 15) and people living with overweight/obesity (*n* = 51) with or without type 2 diabetes. Statistical tests used: Spearman correlation.

To investigate whether ASK1 induces *FGF21* expression in humans, we analyzed hepatic *ASK1* and *FGF21* mRNA expression in lean study participants and people living with obesity with or without type 2 diabetes. Basic clinical characteristics of these participants are provided in Table S1. As shown in Figure 6A, we found a significant positive correlation between liver *ASK1* and *FGF21* expression. Sex‐specific analysis revealed that this positive correlation is found in both women and men (Figure S6A,B). In addition, *ASK1* expression correlated negatively with BMI (Figure 6B), confirming previous findings in a smaller cohort [20]. In support of a protective role of liver‐expressed ASK1 in the development of human adiposity, *ASK1* expression correlated negatively with visceral fat area determined by abdominal CT or MRI scans (Figure 6C), a finding that remained significant in subpopulation analysis of both sexes (Figure S6C,D). Moreover, liver *FGF21* expression correlated negatively with visceral fat area (Figure 6D), suggesting a potential link between ASK1‐induced *FGF21* expression and reduced (visceral) adiposity in humans.

## 4 Discussion

The present study unravels a yet undescribed role of hepatic ASK1 in the synthesis of FGF21 and, hence, in the regulation of adiposity and metabolic health. In particular, we report that liver‐specific ASK1 overexpression prevented HFD‐induced obesity and associated deterioration of glucose metabolism in mice. This lean and healthy metabolic phenotype is at least partly mediated by FGF21, given that HFD‐fed ASK1^+hep^ mice displayed significantly elevated systemic FGF21 levels and that lowering FGF21 abolished the positive effect on adiposity. In contrast to FGF21, its receptor expression (*Klb*, *Fgfr1*) was not altered in liver, WAT, and BAT of ASK1^+hep^ mice. The fact that systemic FGF21 levels increased significantly in ASK1^+hep^ mice after 7 weeks of HFD feeding (at a time point when WAT mass and body weight were still similar to control mice) suggests that ASK1 overexpression increases FGF21 production independently of changes in body weight or adiposity. The positive effect of ASK1 overexpression on FGF21 synthesis and body weight gain was found in both female and male mice, suggesting a sex‐independent effect. In contrast to HFD‐fed mice, we found no significant effect of ASK1 overexpression on circulating FGF21 concentrations and body weight gain in chow‐fed mice.

Of note, the resistance to diet‐induced obesity in HFD‐fed ASK1^+hep^ mice was paralleled by elevated adipose levels of UCP1 and increased energy expenditure, indicative of an FGF21‐mediated increase in thermogenesis‐driven energy expenditure [5, 14, 15]. Yet we cannot exclude FGF21‐mediated, UCP1‐independent effects affecting energy and glucose metabolism in ASK1^+hep^ mice as has been previously suggested [35, 36, 37]. Moreover, other energy‐consuming processes, such as futile substrate cycling in WAT might additionally contribute to the observed increase in energy expenditure in HFD‐fed ASK1^+hep^ mice. As *Ask1* expression was similar in BAT and WAT of HFD‐fed ASK1^f/f^ and ASK1^+hep^ mice, increased thermogenesis in the latter may not be mediated by changes in ASK1 levels in adipose tissue [38, 39]. In support of an important role of the ASK1‐FGF21 axis in the development of human adiposity, hepatic *ASK1* expression correlated positively with *FGF21* but negatively with visceral WAT area. Similar to the finding in rodents, such correlations were found in both female and male individuals.

ATF4 is an adaptive response regulator of metabolic homeostasis and plays a key role in the adaptation to cellular stress [40]. In particular, ATF4 is a regulator of *Fgf21* gene transcription in response to nutritional stimuli such as HFD [41]. Herein, we provide evidence that ATF4 is critically involved in ASK1 overexpression‐mediated *Fgf21* gene expression. Indeed, in silico analysis of bulk RNA sequencing data predicted ATF4 as one of the top candidates contributing to transcriptional changes observed in livers of HFD‐fed ASK1^+hep^ mice. Moreover, silencing *Atf4* in primary hepatocytes isolated from these mice significantly reduced *Fgf21* transcript levels.

Interestingly, an interaction between the MAP kinase p38, a downstream kinase of ASK1, and ATF4 has been previously described [32, 42]. Since inhibition of p38 reduced *Fgf21* expression to a higher extent in primary hepatocytes isolated from HFD‐fed ASK1^f/f^ compared to ASK1^+hep^ mice, we postulate that ASK1 overexpression activated the p38‐ATF4 axis, which in turn induces *Fgf21* gene expression. Of note, the finding that ASK1 can induce *FGF21* expression is not novel. In 2021, Ogawa et al. reported that ASK1‐p38 signaling induces FGF21 to promote mechanical cell competition and motility in canine kidney cells [43].

In contrast to our findings, Xiang et al. found that liver‐specific ASK1 overexpressing mice exhibited aggravated HFD‐induced obesity compared to controls [44]. While constitutive liver‐specific ASK1 overexpression yielded a ~2‐fold increase in both mouse models, Xiang et al. overexpressed constitutively kinase‐active ASK1, which was not the case in our mouse model [20, 44]. Rather, activity of ASK1 in ASK1^+hep^ mice may be subjected to environmental and/or metabolic cues such as light phase or food intake. Accordingly, ASK1 activity may fluctuate in a circadian manner in these animals but not in mice overexpressing kinase‐active ASK1, potentially explaining opposing findings.

FGF21 has gained scientific interest as a potential candidate in the treatment of obesity and associated metabolic complications [9, 11, 15, 45, 46]. Indeed, direct or GDF15‐mediated overexpression of hepatic *Fgf21* reduced HFD‐induced obesity and MASLD in mice [47, 48]. In line, adipocyte‐specific overexpression of FGF21 improved metabolic health in HFD‐fed mice [49]. Moreover, there are ongoing efforts to increase circulating FGF21 levels by injecting FGF21 analogues or FGF21 receptor agonist to reduce body weight and improve metabolic health in humans [40]. However, while this strategy works reasonably well in mice, it has had limited success in obese nonhuman primates and humans living with obesity, mainly due to adverse side effects and short half‐life times [50, 51]. Herein, we have generated a mouse model in which overexpression of the stress kinase ASK1 in the liver leads to endogenously elevated FGF21 concentrations. Continuous stimulation of endogenous FGF21 synthesis may be a more promising strategy than elevating circulating FGF21 via exogenous administration of a recombinant protein, possibly circumventing drawbacks of the latter. We hypothesize that this concept allows us to avoid two main limitations of exogenously elevated FGF21 concentrations: adverse side effects such as elevation of heart rate/blood pressure and gastrointestinal disorders [46, 52] as well as high injection frequency provoked by the short half‐life time of FGF21 [51].

In conclusion, our studies unravel a novel role of hepatic ASK1 in the synthesis of FGF21, which positively affects energy and glucose metabolism. Hence, increasing hepatic *ASK1* expression might be a novel strategy to improve metabolic health.

## Author Contributions

A.G. designed and performed experiments, analyzed data, and wrote the manuscript. T.D.C. designed and performed experiments and analyzed data. C.V.‐.V., M.B. and P.P.K. performed experiments. C.W. gave conceptual advice and supervised experiments. M.B. performed experiments and gave conceptual advice. S.W. designed and performed experiments, analyzed data, and wrote the manuscript. D.K. designed experiments, analyzed data, and wrote the manuscript. All authors reviewed and commented on the manuscript.

## Funding

This work was supported by the Swiss National Science Foundation (#310030_179344 and 310030_215451 to D.K.), the Uniscientia foundation (#208‐2023 to D.K.), the “Stiftung für wissenschaftliche Forschung an der Universität Zürich” (STWF‐23‐008 to D.K.), and the Children's Research Centre (to A.G.).

## Conflicts of Interest

M.B. received honoraria as a consultant and speaker from Abbott, Amgen, AstraZeneca, Bayer, Boehringer Ingelheim, Daiichi‐Sankyo, Lilly, MSD, Novo Nordisk, Novartis, and Sanofi. All other authors declare no conflicts of interest.

## Supporting information


**Table S1:** Clinical characteristics of human study participants.

**Figure S1:** Similar body weight gain and glucose metabolism in chow‐fed ASK1^f/f^ and ASK1^+hep^ mice. (A) Body weight development of chow‐fed ASK1^f/f^ and ASK1^+hep^ mice (*n* = 6 mice per group). (B). BAT and WAT depot weight normalized to body weight (perigonadal [peri, **p = 0.0011], retroperitoneal [retro, ***p = 0.0003], inguinal [ing, ***p = 0.0006]) of 20 weeks HFD‐fed ASK1^+hep^ (blue bar) and ASK1^f/f^ (black bar) (*n* = 10–11 mice). Student's *t* test. Plasma glucose (C) and insulin levels (D) after 5 h fasting in ASK1^+hep^ and ASK1^f/f^ mice on HFD for 20 weeks (*n* = 10–11 mice). ipGTT (E; *n* = 6–10 mice) and ipITT (F; *n* = 5–7 mice) in ASK1^+hep^ and ASK1^f/f^ mice fed a chow diet for 20 weeks. *p = 0.0165 (C), *p = 0.0169 (D) (Mann–Whitney test). Data are shown as mean ± SEM.
**Figure S2:** Similar locomotor activity between HFD‐fed ASK1^f/f^ and ASK1^+hep^ mice. (A) Energy expenditure of ASK1^+hep^ (blue bars/lines) and ASK1^f/f^ (black bars/lines) in mice on HFD for 7 weeks during light phase and dark phase (*n* = 8–9 mice). (B) Locomotor activity of ASK1^+hep^ (blue bar) and ASK1^f/f^ (black bar) in mice on HFD for 7 weeks during light phase and dark phase (*n* = 8–9 mice). (C) Hematoxylin‐ and eosin‐stained sections in BAT harvested from ASK1^f/f^ and ASK1^+hep^ mice fed a HFD for 7 weeks. Data are shown as mean ± SEM.
**Figure S3:** Increased FGF21 concentrations and reduced body weight gain in female HFD‐fed ASK1^+hep^ mice. Body weight (A) and WAT weight (B) of ASK1^f/f^ and ASK1^+hep^ male mice fed a HFD for 7 weeks (*n* = 8–11 mice). C. Relative Klb and Fgfr1 gene expression in livers of 7 weeks HFD‐fed ASK1^+hep^ and ASK1^f/f^ mice (*n* = 5 mice). Relative Ask1, Klb and Fgfr1 gene expression in BAT (D) and WAT (E) of 7 weeks HFD‐fed ASK1^+hep^ and ASK1^f/f^ mice (*n* = 7–9 mice). (F) FGF21 plasma concentrations in 26 weeks old chow‐fed male ASK1^+hep^ and ASK1^f/f^ mice (*n* = 5–13). (G) Circulating FGF21 levels in female HFD‐fed ASK1^+hep^ (blue bars) and ASK1^f/f^ (black bars) mice after 7 weeks on HFD (*n* = 5–8 mice per group). *p = 0.0496 (Student's *t* test). (H) Body weight gain in female HFD‐fed ASK1^+hep^ (blue symbols) and ASK1^f/f^ (black symbols) mice (*n* = 8–9 mice). Data are shown as mean ± SEM.
**Figure S4:** Increased Ask1 expression in primary hepatocytes of HFD‐fed ASK1^+hep^ mice. Relative Fgf21 gene expression after treating primary hepatocytes isolated from 7 weeks HFD‐fed ASK1^+hep^ and ASK1^f/f^ mice with the p38 inhibitor SB203580 (A) or the JNK inhibitor SP600125 (B) for 24 h (*n* = 5–6 culture wells from 2 independent experiments with one mouse per genotype and 2–3 technical replicates per condition). *Atf4* (C) and *Ask1* (D) gene expression after siRNA‐mediated knockdown of ATF4 in primary hepatocytes isolated from 7 weeks HFD‐fed ASK1^+hep^ and ASK1^f/f^ mice (*n* = 7 cell culture wells from 3 independent experiments with one mouse per genotype and 2–3 technical replicates per condition). Data are shown as mean ± SEM. *p < 0.05, **p < 0.01, **** *p* < 0.0001 (two‐way ANOVA).
**Figure S5:** Reduced circulating FGF21 concentrations in AAV shFgf21 treated mice after 20 weeks of HFD. (A) Circulating FGF21 levels in ASK1^+hep^ (blue bars, *n* = 5–8 mice) and ASK1^f/f^ (gray/black bars; *n* = 7–9) mice injected with AAV shCo or AAV shFgf21 and fed a HFD for 20 weeks. *p = 0.0104, ****p < 0.0001 (two‐way ANOVA). (B). WAT weight (perigonadal [peri, #p = 0.0517]), retroperitoneal (retro, *p = 0.0395), inguinal (ing, **p = 0.0058) and BAT (*p = 0.0281) of 20 weeks HFD‐fed ASK1^+hep^ (blue bar) and ASK1^f/f^ (black bar) (*n* = 8–9 mice) injected with AAV shCo. Student's *t* test for periWAT, retroWAT and ingWAT, Mann–Whitney for BAT. (C) WAT and BAT weights of 20 weeks HFD‐fed ASK1^+hep^ (blue bar) and ASK1^f/f^ (gray bar) (*n* = 5–7 mice) injected with AAV shFgf21. Data are shown as mean ± SEM.
**Figure S6:** Liver *ASK1* expression correlates positively with *FGF21* in women and men. Scatterplot of liver *ASK1* mRNA expression and liver *FGF21* mRNA expression in women (A) (*n* = 47) and men (B) (*n* = 49). Scatterplot of liver *ASK1* mRNA expression and visceral fat area (cm^2^) in women (C) (*n* = 29) and men (D) (*n* = 37). Statistical tests used: Spearman correlation.

## Data Availability

All data and uncropped Western blots are included in the source data files. RNA sequencing data have been deposited in Gene Expression Omnibus (GEO) under accession number GSE298858.
