# Supplementary material for: ASK1‐Induced FGF21 Synthesis in the Liver Prevents Obesity in Mice
Source: Obesity (Silver Spring). 2026 Jul 16;34(8):1635–46. doi: 10.1002/oby.70261 (PMC13422662; doi:10.1002/oby.70261)
Supplement: Supplementary file 1 — Table S1: Clinical characteristics of human study participants. [file OBY-34-1635-s002.docx]

**Supplementary Table S1 Clinical characteristics of study participants**

|  | Lean (n=23) | Patients with Overweight/Obesity (n=74) |
| --- | --- | --- |
| Age (years) | 69.8 ± 13.6 | 70.1 ± 14.4 |
| Men/women (n) | 14/9 | 35/39 |
| Type 2 diabetes (n) | 3 | 39 |
| Body mass index (kg/m²) | 24.3 ± 0.2 | 40.0 ± 1.2*** |
| Body fat (%) | 22.3 ± 0.7 | 39.5 ± 1.1*** |
| Visceral fat area^#^ (cm²) | 52.3 ± 4.6 | 213.4 ± 10.1*** |
| Fasting plasma glucose (mmol/l) | 5.4 ± 0.1 | 6.1 ± 0.2** |
| Fasting plasma insulin (pmol/l) | 40 ± 16 | 210 ± 15*** |
| HbA1c (%) | 5.4 ± 0.1 | 6.1 ± 0.1*** |
| Total cholesterol (mmol/l) | 4.8 ± 0.2 | 5.4 ± 0.1** |
| Triglycerides (mmol/l) | 1.1 ± 0.1 | 2.1 ± 0.1*** |
| Free fatty acids (mmol/l) | 0.33 ± 0.06 | 0.59 ± 0.03*** |

Values are expressed as mean ± SEM. **p<0.01, ***p<0.001 (Student’s *t*-test).

^#^data from lean (n=15) and people living with obesity (n=51)
