# Supplementary material for: ASK1‐Induced FGF21 Synthesis in the Liver Prevents Obesity in Mice
Source: Obesity (Silver Spring). 2026 Jul 16;34(8):1635–46. doi: 10.1002/oby.70261 (PMC13422662; doi:10.1002/oby.70261)
Supplement: Supplementary file 2 — Figure S1: Similar body weight gain and glucose metabolism in chow‐fed ASK1f/f and ASK1+hep mice. (A) Body weight development of chow‐fed ASK1f/f and ASK1+hep mice (n = 6 mice per group). (B). BAT and WAT depot weight normalized to body weight (perigonadal [peri, **p = 0.0011], retroperitoneal [retro, ***p = 0.0003], inguinal [ing, ***p = 0.0006]) of 20 weeks HFD‐fed ASK1+hep (blue bar) and ASK1f/f (black bar) (n = 10–11 mice). Student's t test. Plasma glucose (C) and insulin levels (D) after 5 h fasting in ASK1+hep and ASK1f/f mice on HFD for 20 weeks (n = 10–11 mice). ipGTT (E; n = 6–10 mice) and ipITT (F; n = 5–7 mice) in ASK1+hep and ASK1f/f mice fed a chow diet for 20 weeks. *p = 0.0165 (C), *p = 0.0169 (D) (Mann–Whitney test). Data are shown as mean ± SEM. Figure S2: Similar locomotor activity between HFD‐fed ASK1f/f and ASK1+hep mice. (A) Energy expenditure of ASK1+hep (blue bars/lines) and ASK1f/f (black bars/lines) in mice on HFD for 7 weeks during light phase and dark phase (n = 8–9 mice). (B) Locomotor activity of ASK1+hep (blue bar) and ASK1f/f (black bar) in mice on HFD for 7 weeks during light phase and dark phase (n = 8–9 mice). (C) Hematoxylin‐ and eosin‐stained sections in BAT harvested from ASK1f/f and ASK1+hep mice fed a HFD for 7 weeks. Data are shown as mean ± SEM. Figure S3: Increased FGF21 concentrations and reduced body weight gain in female HFD‐fed ASK1+hep mice. Body weight (A) and WAT weight (B) of ASK1f/f and ASK1+hep male mice fed a HFD for 7 weeks (n = 8–11 mice). C. Relative Klb and Fgfr1 gene expression in livers of 7 weeks HFD‐fed ASK1+hep and ASK1f/f mice (n = 5 mice). Relative Ask1, Klb and Fgfr1 gene expression in BAT (D) and WAT (E) of 7 weeks HFD‐fed ASK1+hep and ASK1f/f mice (n = 7–9 mice). (F) FGF21 plasma concentrations in 26 weeks old chow‐fed male ASK1+hep and ASK1f/f mice (n = 5–13). (G) Circulating FGF21 levels in female HFD‐fed ASK1+hep (blue bars) and ASK1f/f (black bars) mice after 7 weeks on HFD (n = 5–8 mice per grou [file OBY-34-1635-s001.pptx]

## Slide 1
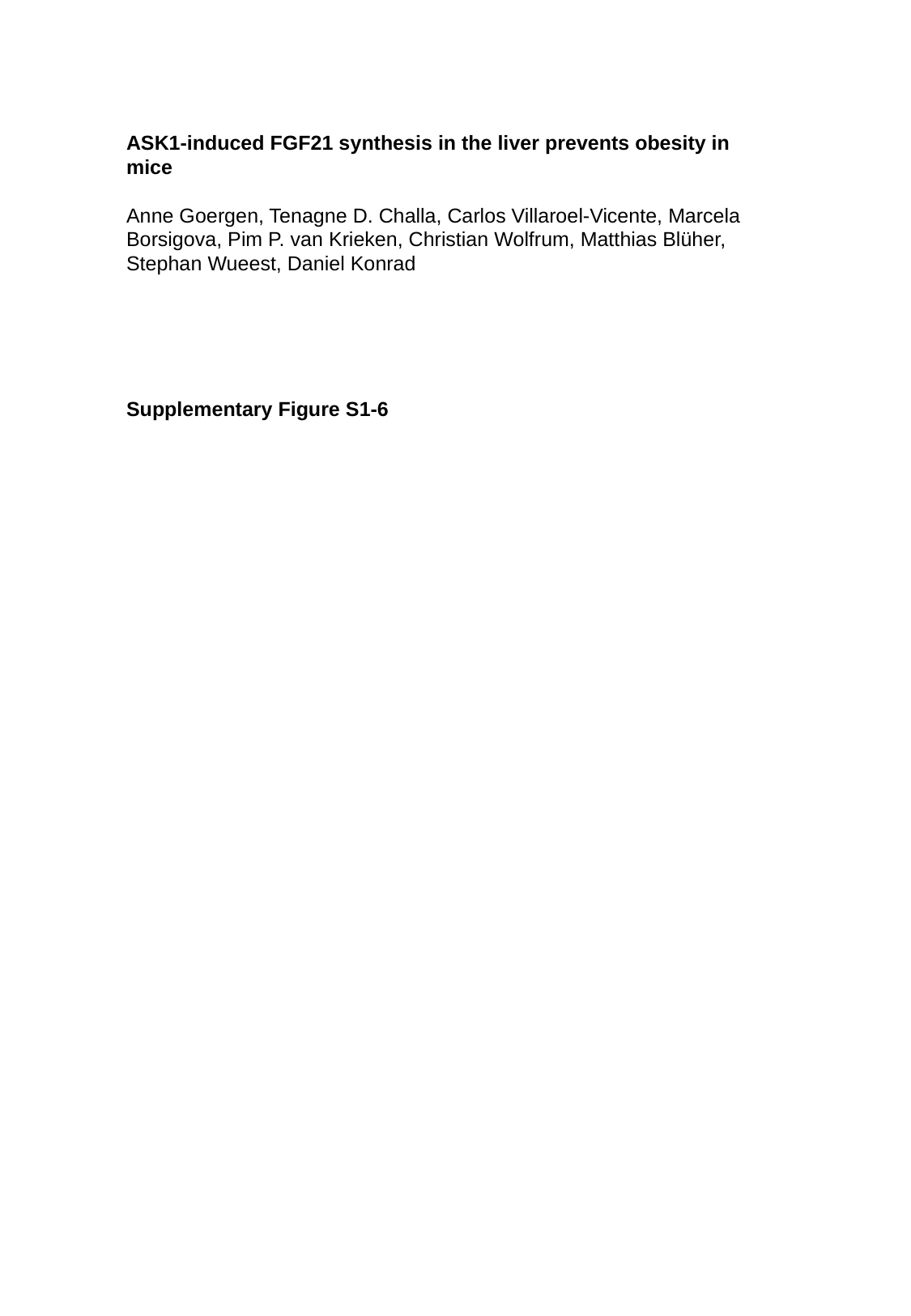

ASK1-induced FGF21 synthesis in the liver prevents obesity in mice
Anne Goergen, Tenagne D. Challa, Carlos Villaroel-Vicente, Marcela Borsigova, Pim P. van Krieken, Christian Wolfrum, Matthias Blüher, Stephan Wueest, Daniel Konrad
Supplementary Figure S1-6

## Slide 2
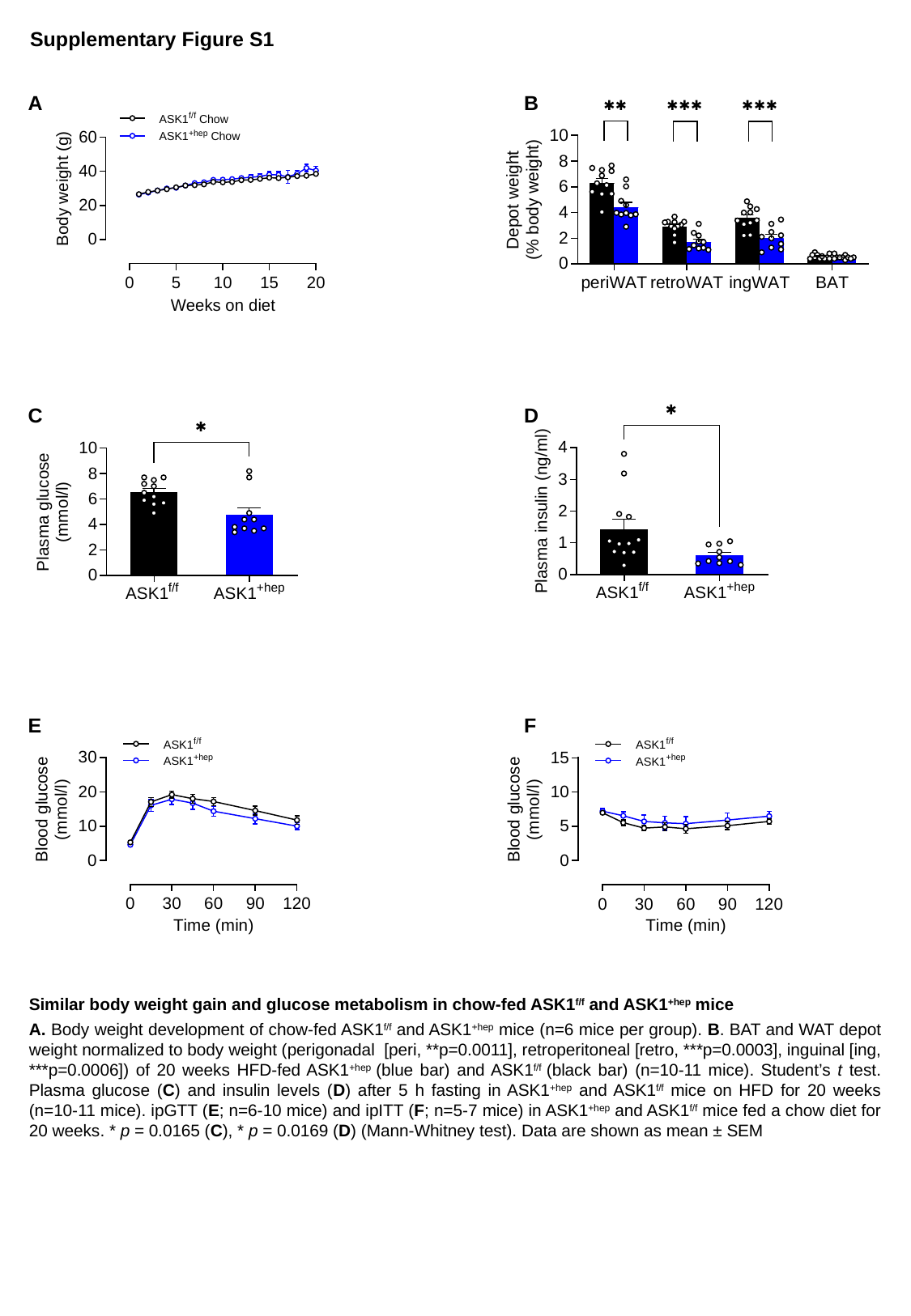

Supplementary Figure S1
A
B
C
D
E
F
Similar body weight gain and glucose metabolism in chow-fed ASK1f/f and ASK1+hep mice
A. Body weight development of chow-fed ASK1f/f and ASK1+hep mice (n=6 mice per group). B. BAT and WAT depot weight normalized to body weight (perigonadal [peri, **p=0.0011], retroperitoneal [retro, ***p=0.0003], inguinal [ing, ***p=0.0006]) of 20 weeks HFD-fed ASK1+hep (blue bar) and ASK1f/f (black bar) (n=10-11 mice). Student’s t test. Plasma glucose (C) and insulin levels (D) after 5 h fasting in ASK1+hep and ASK1f/f mice on HFD for 20 weeks (n=10-11 mice). ipGTT (E; n=6-10 mice) and ipITT (F; n=5-7 mice) in ASK1+hep and ASK1f/f mice fed a chow diet for 20 weeks. * p = 0.0165 (C), * p = 0.0169 (D) (Mann-Whitney test). Data are shown as mean ± SEM

## Slide 3
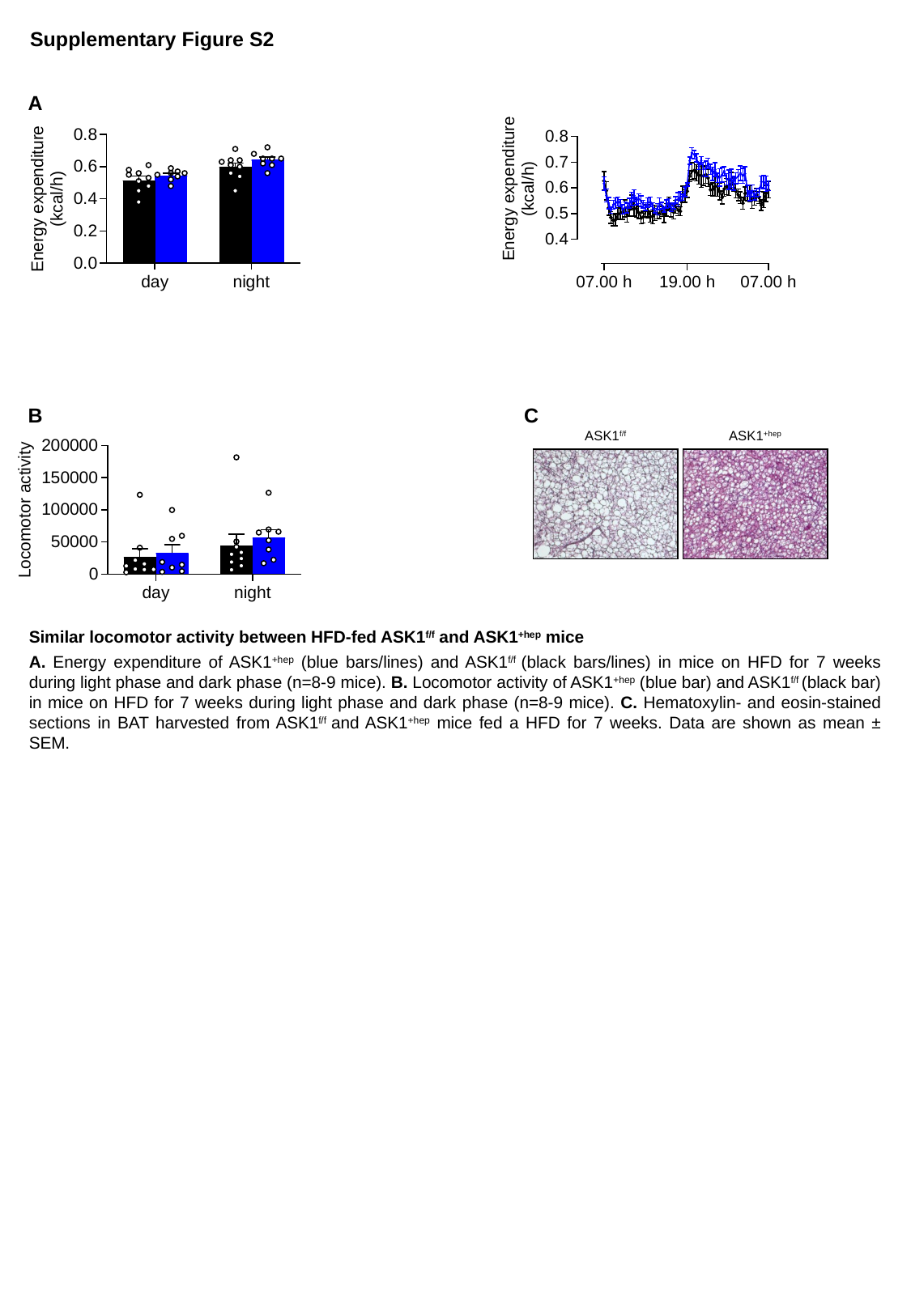

Supplementary Figure S2
A
B
C
ASK1f/f
ASK1+hep
Similar locomotor activity between HFD-fed ASK1f/f and ASK1+hep mice
A. Energy expenditure of ASK1+hep (blue bars/lines) and ASK1f/f (black bars/lines) in mice on HFD for 7 weeks during light phase and dark phase (n=8-9 mice). B. Locomotor activity of ASK1+hep (blue bar) and ASK1f/f (black bar) in mice on HFD for 7 weeks during light phase and dark phase (n=8-9 mice). C. Hematoxylin- and eosin-stained sections in BAT harvested from ASK1f/f and ASK1+hep mice fed a HFD for 7 weeks. Data are shown as mean ± SEM.

## Slide 4
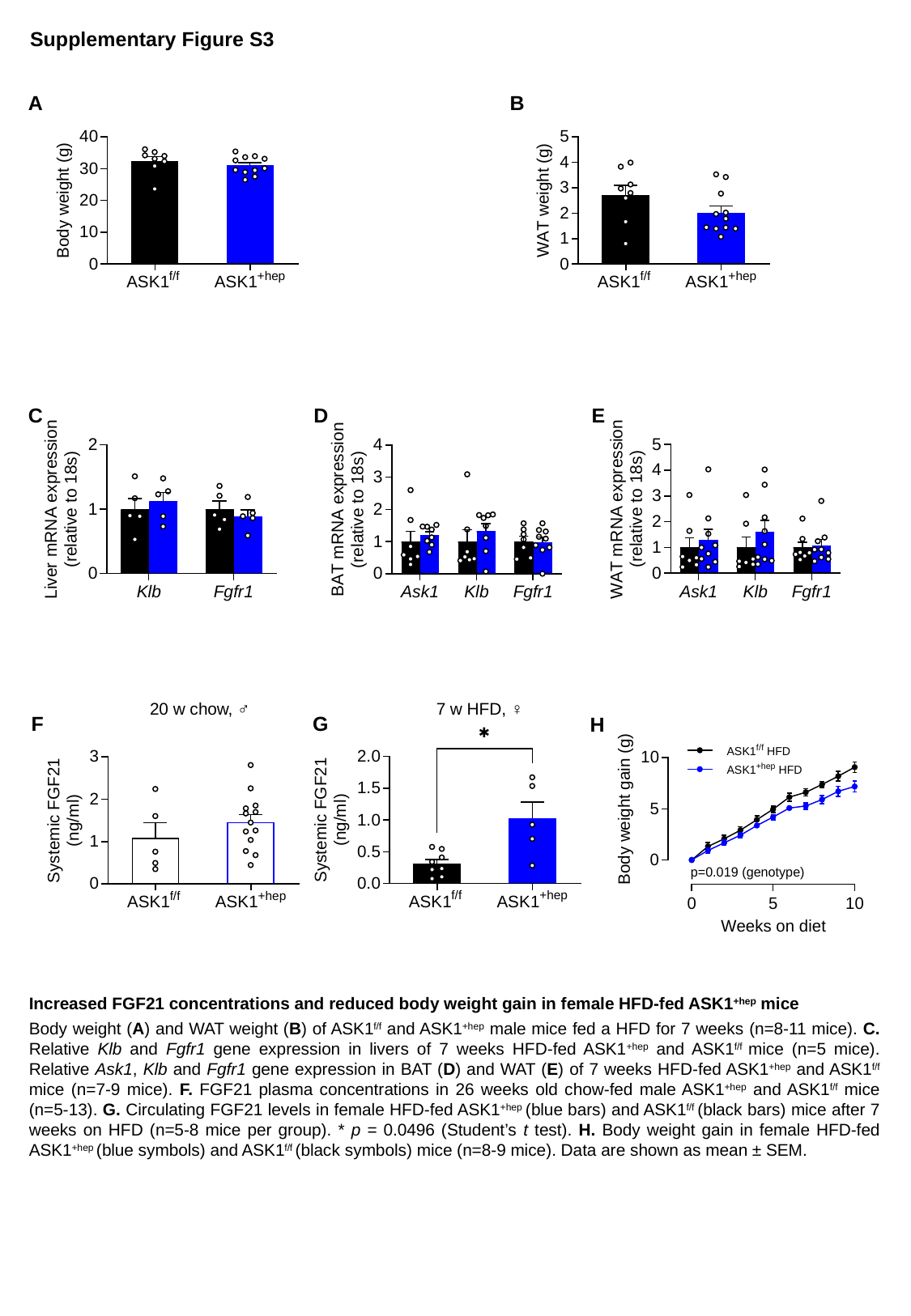

Supplementary Figure S3
A
B
C
D
E
20 w chow, ♂
7 w HFD, ♀
F
G
H
Increased FGF21 concentrations and reduced body weight gain in female HFD-fed ASK1+hep mice
Body weight (A) and WAT weight (B) of ASK1f/f and ASK1+hep male mice fed a HFD for 7 weeks (n=8-11 mice). C. Relative Klb and Fgfr1 gene expression in livers of 7 weeks HFD-fed ASK1+hep and ASK1f/f mice (n=5 mice). Relative Ask1, Klb and Fgfr1 gene expression in BAT (D) and WAT (E) of 7 weeks HFD-fed ASK1+hep and ASK1f/f mice (n=7-9 mice). F. FGF21 plasma concentrations in 26 weeks old chow-fed male ASK1+hep and ASK1f/f mice (n=5-13). G. Circulating FGF21 levels in female HFD-fed ASK1+hep (blue bars) and ASK1f/f (black bars) mice after 7 weeks on HFD (n=5-8 mice per group). * p = 0.0496 (Student’s t test). H. Body weight gain in female HFD-fed ASK1+hep (blue symbols) and ASK1f/f (black symbols) mice (n=8-9 mice). Data are shown as mean ± SEM.

## Slide 5
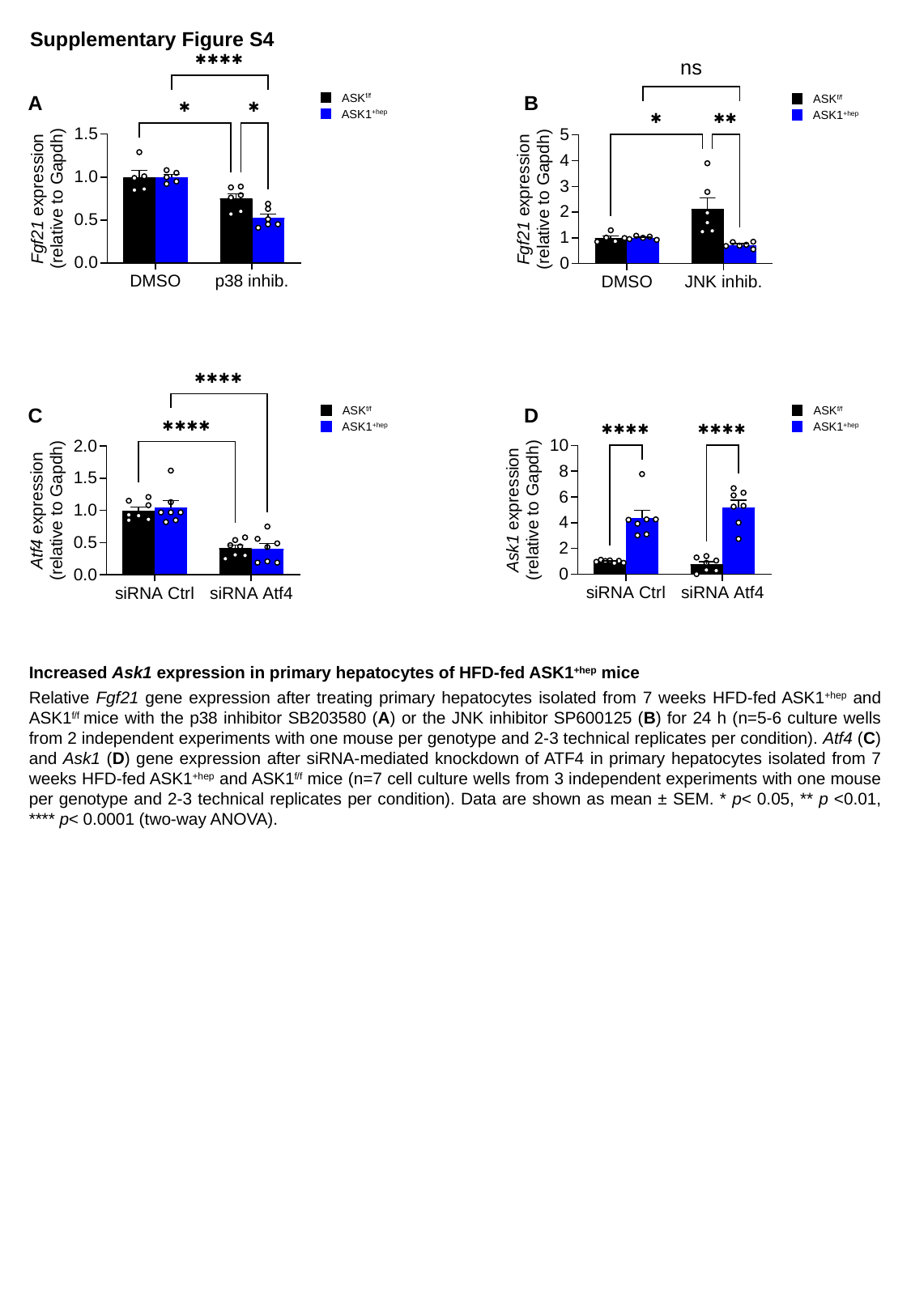

Supplementary Figure S4
A
B
ASKf/f
ASK1+hep
ASKf/f
ASK1+hep
C
D
ASKf/f
ASK1+hep
ASKf/f
ASK1+hep
Increased Ask1 expression in primary hepatocytes of HFD-fed ASK1+hep mice
Relative Fgf21 gene expression after treating primary hepatocytes isolated from 7 weeks HFD-fed ASK1+hep and ASK1f/f mice with the p38 inhibitor SB203580 (A) or the JNK inhibitor SP600125 (B) for 24 h (n=5-6 culture wells from 2 independent experiments with one mouse per genotype and 2-3 technical replicates per condition). Atf4 (C) and Ask1 (D) gene expression after siRNA-mediated knockdown of ATF4 in primary hepatocytes isolated from 7 weeks HFD-fed ASK1+hep and ASK1f/f mice (n=7 cell culture wells from 3 independent experiments with one mouse per genotype and 2-3 technical replicates per condition). Data are shown as mean ± SEM. * p< 0.05, ** p <0.01, **** p< 0.0001 (two-way ANOVA).

## Slide 6
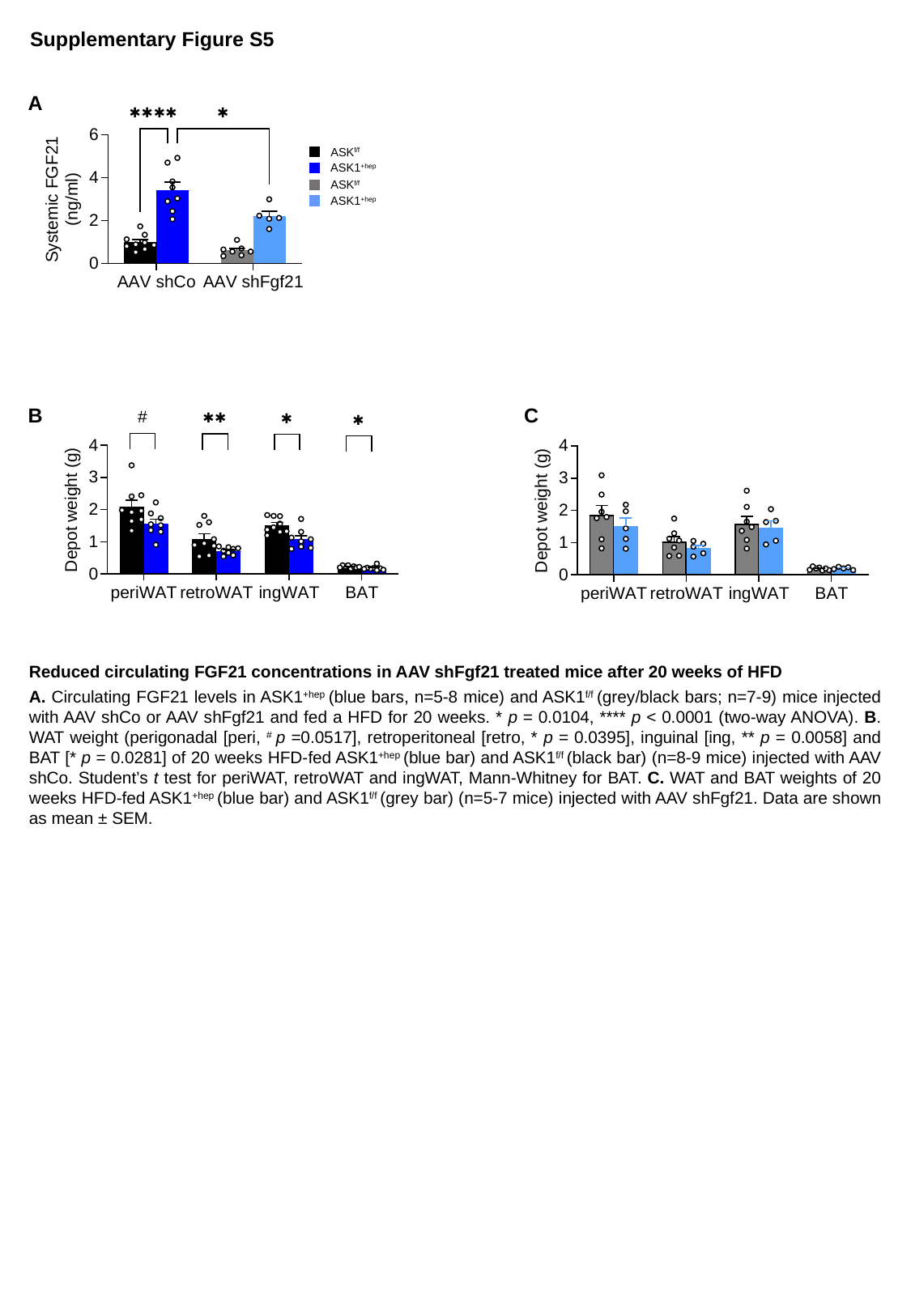

Supplementary Figure S5
A
ASKf/f
ASK1+hep
ASKf/f
ASK1+hep
B
C
Reduced circulating FGF21 concentrations in AAV shFgf21 treated mice after 20 weeks of HFD
A. Circulating FGF21 levels in ASK1+hep (blue bars, n=5-8 mice) and ASK1f/f (grey/black bars; n=7-9) mice injected with AAV shCo or AAV shFgf21 and fed a HFD for 20 weeks. * p = 0.0104, **** p < 0.0001 (two-way ANOVA). B. WAT weight (perigonadal [peri, # p =0.0517], retroperitoneal [retro, * p = 0.0395], inguinal [ing, ** p = 0.0058] and BAT [* p = 0.0281] of 20 weeks HFD-fed ASK1+hep (blue bar) and ASK1f/f (black bar) (n=8-9 mice) injected with AAV shCo. Student’s t test for periWAT, retroWAT and ingWAT, Mann-Whitney for BAT. C. WAT and BAT weights of 20 weeks HFD-fed ASK1+hep (blue bar) and ASK1f/f (grey bar) (n=5-7 mice) injected with AAV shFgf21. Data are shown as mean ± SEM.

## Slide 7
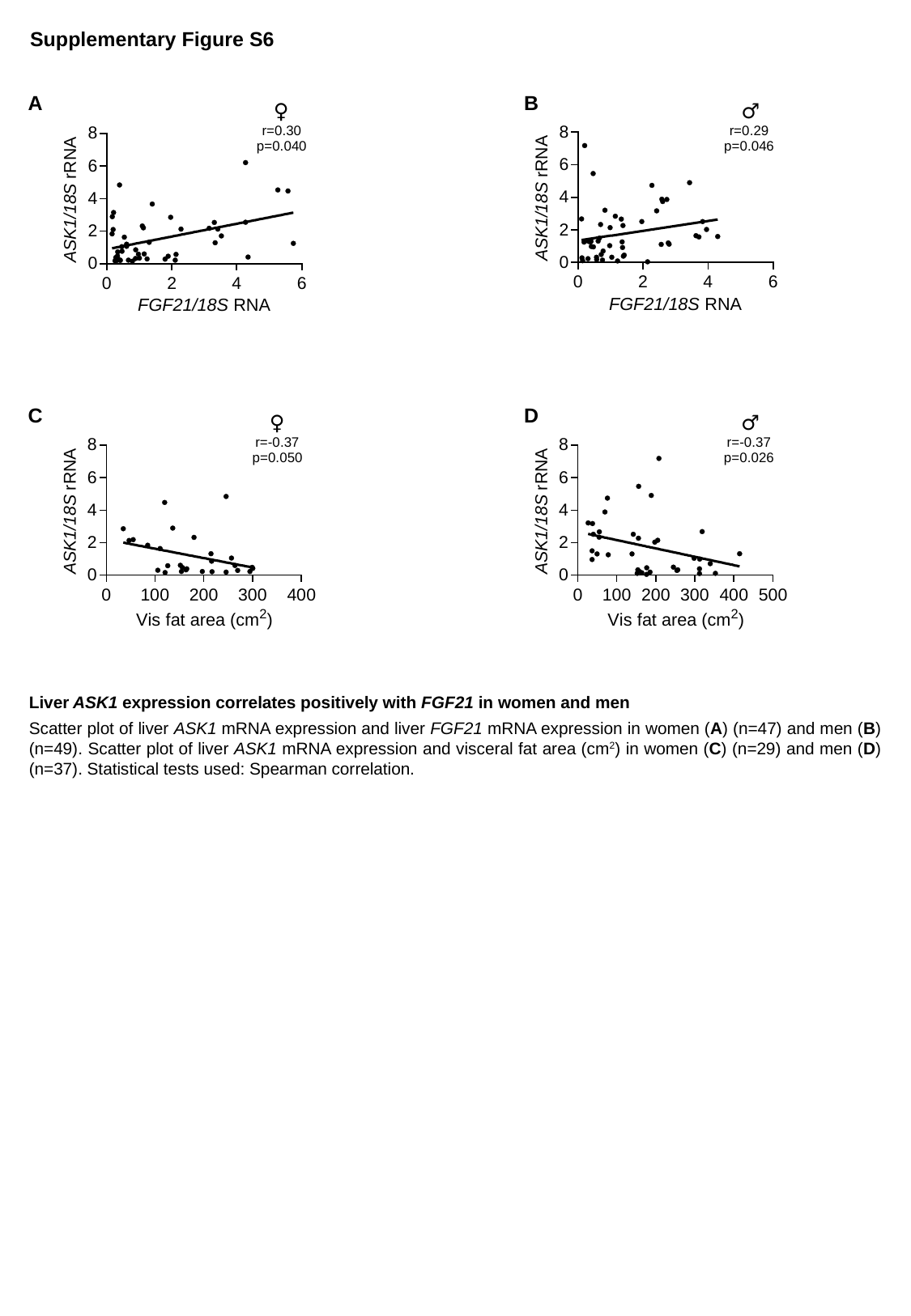

Supplementary Figure S6
A
B
C
D
Liver ASK1 expression correlates positively with FGF21 in women and men
Scatter plot of liver ASK1 mRNA expression and liver FGF21 mRNA expression in women (A) (n=47) and men (B) (n=49). Scatter plot of liver ASK1 mRNA expression and visceral fat area (cm2) in women (C) (n=29) and men (D) (n=37). Statistical tests used: Spearman correlation.
